# Emergent Functional Properties of Neuronal Networks with Controlled Topology

**DOI:** 10.1371/journal.pone.0034648

**Published:** 2012-04-06

**Authors:** Emanuele Marconi, Thierry Nieus, Alessandro Maccione, Pierluigi Valente, Alessandro Simi, Mirko Messa, Silvia Dante, Pietro Baldelli, Luca Berdondini, Fabio Benfenati

**Affiliations:** 1 Department of Neuroscience and Brain Technologies, Fondazione Istituto Italiano di Tecnologia, Genova, Italy; 2 Department of Nanophysics, Fondazione Istituto Italiano di Tecnologia, Genova, Italy; 3 Department of Experimental Medicine, Section of Physiology, University of Genoa, Genova, Italy; University of Michigan, United States of America

## Abstract

The interplay between anatomical connectivity and dynamics in neural networks plays a key role in the functional properties of the brain and in the associated connectivity changes induced by neural diseases. However, a detailed experimental investigation of this interplay at both cellular and population scales in the living brain is limited by accessibility. Alternatively, to investigate the basic operational principles with morphological, electrophysiological and computational methods, the activity emerging from large *in vitro* networks of primary neurons organized with imposed topologies can be studied. Here, we validated the use of a new bio-printing approach, which effectively maintains the topology of hippocampal cultures *in vitro* and investigated, by patch-clamp and MEA electrophysiology, the emerging functional properties of these grid-confined networks. In spite of differences in the organization of physical connectivity, our bio-patterned grid networks retained the key properties of synaptic transmission, short-term plasticity and overall network activity with respect to random networks. Interestingly, the imposed grid topology resulted in a reinforcement of functional connections along orthogonal directions, shorter connectivity links and a greatly increased spiking probability in response to focal stimulation. These results clearly demonstrate that reliable functional studies can nowadays be performed on large neuronal networks in the presence of sustained changes in the physical network connectivity.

## Introduction

Understanding the interplay between anatomical connectivity and dynamics is relevant to unravel the underlying operational principles in complex neuronal systems. At the micro-circuit level, detailed descriptions of distinct types of neuronal connectome have been reported [1], [2], indicating both cell-specific roles associated with the balance between excitatory and inhibitory neurons [3] and a hierarchical “small world” (or scale-free) connectivity organization [4]–[6], which includes superconnected nodes with mainly short range connections and a small number of long-range connections [7]. At the subcellular level, changes in synaptic connectivity results in circuit refinement and changes in the efficacy of synaptic connections [8].

Theoretical studies have recently provided the proper mathematical tools to classify neural networks based on their connectivity patterns [9]. The formal approach, called Graph Theory, affords the categorization of the topologies (i.e. random, regular and small word networks) based on statistical coefficients computed from the connectivity patterns such as the clustering coefficient or the mean path-length [7]. By using these tools, various computational studies have shown how network synchrony states in complex networks are related to the underlying topology [10]–[12], in an attempt to link the network topology to the activity expressed by hierarchically-organized excitable networks [13]. Interestingly, neurological diseases such as epilepsy [14] or Alzheimer's disease [15] have been associated with changes in network topology and functional connectivity.

A promising methodological approach to investigate the basic principles of the physical and functional connectivity within large neuronal networks consists in studying the activity emerging from living networks with imposed topologies. This can be implemented by the combined use of bio-patterning technologies to spatially control neuronal network growth and microelectrode arrays (MEAs) for achieving a long-term, non-invasive neuroelectronic interfacing [16]–[18]. However, in order to reach the full maturation of the network, this approach requires the preservation of the spatial confinement of neuronal structures over time (i.e., more than 3 weeks in vitro [19]–[20]). This critical issue was investigated using several approaches [16]–[21], which however displayed significant drawbacks [22]–[40]. Hanein and co-authors [38] recently presented a method based on the use of carbon nanotubes to growth neuronal islands in correspondence with the microelectrode sites. However, this method does not provide any control on the morphology of the inter-islands connections.

In this work, we achieved patterning and growth of neuronal cultures for more than 20 days *in vitro* (DIV) by coupling the micro-contact printing of an adhesion promoter with the use of an agarose repulsive layer [41], [42] and investigated the electrophysiological features of these preparations at both synaptic and network levels with respect to random cultures. We found that, the basic properties of synaptic transmission, the overall network development and the emerging overall network activity were not altered with respect to control random cultures. Notably, the grid topology imposed to the networks was associated with a reinforcement of functional connectivity along orthogonal directions, shorter connectivity links and an increased spiking probability in response to focal stimulation.

## Materials and Methods

### Preparation of the cell culture substrates

All procedures involving experimental animals were approved by the institutional IIT ethic committee and by the Italian Ministry of Health and Animal Care (authorization ID 227, prot. 4127 March 25, 2008). Random and bio-patterned primary neuronal cultures were grown on glass coverslips (0.13–0.16 mm in thickness, Menzel-Gläser, Braunschweig, Germany) or MEAs (8×8 TiN/SiN MEAs with electrodes of 30 µm in diameter and 200 µm pitch, from Multi Channel Systems, Reutlingen, Germany). Substrates for random cultures were treated with a Poly-D-Lysine (PDL; 70–150 kDa, Sigma-Aldrich, St Louis, MO, USA) coating solution (Poly-D-Lysine - PDL - 0.1 mg/mL in double-distilled water (DDW)) and maintained overnight at 37°C with saturated humidity and 5% CO_2_ before cell seeding. Glass coverslips and MEAs for bio-patterned networks were treated with UV/Ozone ProCleaner (BioForce Nanosciences Inc., Ames, IA, USA) for 45 min to improve the surface hydrophilicity, coated with type IIA agarose (0.15% w/v in DDW, Sigma-Aldrich), stored at 4°C and subjected to micro-contact printing within 2 days.

**Figure 1:**
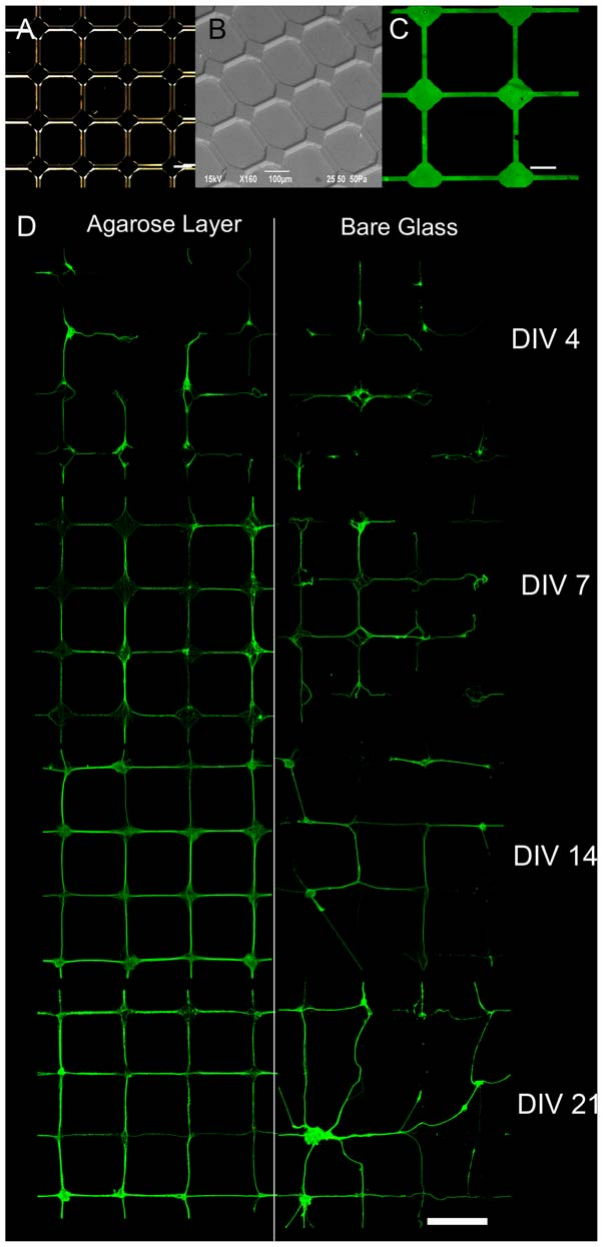
Overview of micro-contact printing tools and patterned cultures. *(A)* Microphotograph of the Si-etched master. *(B)* ESEM picture of an area of the PDMS stamp replicated from the Si master. *(C)* Fluorescence image of a representative FITC-PLL printed area on a glass coverslip. Scalebar: *A* and *C*, 50 µm; *B*, 100 µm. *(D)* Preservation of the bio-printed pattern on either agarose-coated (*left panels*) or uncoated (*right panels*) glass coverslips as a function of days in vitro (*DIV*). E18 rat hippocampal neurons were stained with an anti-βTubIII mAb. The use of the agarose layer to inhibit cellular adhesion preserved the printed grid topology of primary neurons over time. Scalebar: 200 µm.

**Micro-contact printing**: Silicon masters were produced by standard photolithographic techniques and Si wet etching (KOH) at MDM-INFM laboratories (Agrate Brianza, Milano, Italy). The silicon surface (1 cm^2^) was structured in a grid pattern (5 µm wide lines and 200 µm interline spacing; see Fig. 1*A*). Polydimethyl siloxane (PDMS) stamps were cast from the masters, using Sylgard 184 silicone elastomer (Dow Corning, Midland, MI). The elastomer was combined with the curing agent at 10∶1 ratio, poured onto the masters and degassed. PDMS stamps were subsequently baked at 120°C for 12 h, removed from Si masters and stored for at least 1 day in DDW before use. For printing, PDMS stamps were inked for 5 min in a 1∶100 solution of extracellular matrix (ECM) gel (Sigma-Aldrich) and 0.01 mg/mL PDL in Neurobasal (Gibco-Invitrogen, Carlsbad, CA, USA). Inked stamps were dried under N_2_ and kept in contact with the substrates for 5 min under constant pressure. Stamps were aligned on the active area of MEAs by using a custom printing system providing *xyz* axis movements, rotation and tilt adjustments under a Leica A60 microscope (Leica, Wetzlar, Germany). Finally, the printed substrates were washed with sterile DDW. All bio-printing operations were performed under a sterile hood.

**Neuronal cultures:** Culturing media and additional compounds were acquired from Gibco-Invitrogen. Primary hippocampal neurons were prepared from E18 rat as previously described [21], [43]. Briefly, hippocampi were manually isolated in Hanks' Balanced Salt Solutions (HBSS) and incubated for 15 min with 0.125% trypsin at 37°C. After trypsinization, tissues were mechanically dissociated through a fire-polished Pasteur pipette. Successively, cells were plated at 70 cells/mm^2^ in serum-free Neurobasal supplemented with B27 and incubated at 37°C in 5% CO_2_. To sustain the growth of low-density preparations, cultures on coverslips were grown in multiwell plates (Corning, Lowell, MA, USA) seeded with feeder cultures. Each well was coated with 0.1 mg/mL PDL and paraffin drops were used to elevate coverslips. The bottom of the well was seeded with dense (150 cells/mm^2^) feeder hippocampal cultures. After seeding, coverslips were transferred into the wells and placed on the paraffin drops with the cell culture side facing the feeder culture. For cultures on MEAs, a PDMS structure was realized to support the feeder culture surrounding the electrode area. These PDMS structures were sterilized and coated with 0.1 mg/mL PDL as described for coverslips. Successively, hippocampal neurons were seeded at high density on the PDMS structures (50000 cells in 150 µL Neurobasal) and at low-density on the active MEA area (9000 cells in 50 µL Neurobasal).

**Immunofluorescence protocols:** Cells were washed once in 1× phosphate buffered saline (PBS) solution, fixed with 4% (w/v) paraformaldehyde in PBS at room temperature (RT) for 30 min and washed three times in 1× PBS. Fixed samples were permeabilized with 0.1% (v/v) Triton X-100 in 1× PBS for 15 min. Blocking solution (PBS 1×, 1% BSA, 5% FBS) was added for 45 min at RT to block nonspecific reactions. The following primary antibodies diluted in blocking solution were used: rabbit anti-βTubulin III (βTubIII), mouse anti-GFAP (Millipore, Billerica, MA, USA) and mouse anti-synaptotagmin (Syt; kindly provided by Maurizio Popoli). After incubation for 3 h at RT, samples were washed and incubated with secondary Alexa^488^ labelled goat anti-rabbit or Alexa^546^ labelled goat anti-mouse mAb (Invitrogen) for 45 min at RT. Samples were mounted using Prolong anti-fade reagent containing DAPI (Invitrogen) and stored at 4°C. Confocal and widefield fluorescence microscopy were performed using an SP5 microscope (Leica-Microsystems, Wetzlar, Germany) and a Neurolucida imaging system (Mbf Bioscience, Williston, VT, USA) mounted on an Olympus BX 51 microscope. Images were analysed with ImageJ [44] completed with LOCI plug-in [45] and elaborated with Adobe Photoshop CS3 Suite (Adobe, San Jose, CA, USA).

**Patch-clamp recordings:** Whole-cell patch-clamp recordings were made at RT from neurons grown in random and bio-patterned networks. Excitatory or inhibitory postsynaptic currents (EPSCs or IPSCs) were investigated in 14–21 DIV neurons by using an EPC-10 amplifier (HEKA Electronic, Lambrecht Germany). Patch electrodes, fabricated from thick borosilicate glass (Hilgenberg, Mansfield, Germany), were pulled to a final resistance of 3–4 MΩ. Neurons were voltage clamped at −70 mV and presynaptic stimuli were delivered through a glass pipette (1 µm tip diameter) filled with extracellular solution and placed in contact with a neurite near the patched neuron in a loose seal configuration. Current pulses of variable amplitude (10–45 µA) and lasting 0.1/0.3 ms were delivered by an isolated pulse stimulator (model 2100; A-M Systems, Carlsburg, WA) to evoke postsynaptic currents with short latency (2–4 ms). For the data presented in this paper, the extracellular stimulation was adjusted to the minimal intensity needed to elicit successful single monosynaptic synaptic transmission. Although pair recordings represent the best approach to guarantee activation of single monosynaptic responses, we adopted extracellular stimulation to evaluate whether, in patterned cultures, the minimal stimulation method more easily recruits monosynaptic EPSCs without multiple EPSC peaks in the decay phase. The inhibitory or excitatory nature of the postsynaptic currents was routinely identified applying either bicuculline (30 µM) or 6-cyano-7-nitroquinoxaline-2,3-dione (CNQX, 5 µM) to the extracellular bath. IPSCs and EPSCs were recorded by superfusing the whole-cell clamped postsynaptic neuron with an extracellular Tyrode solution containing (in mM): 2 CaCl_2_, 150 NaCl, 1 MgCl_2_, 10 HEPES, 4 KCl, 10 glucose, pH 7.4. D-(-)-2-amino-5-phosphonopentanoic acid (D-AP5) (50 µM) and CGP58845 (5 µM) (Tocris, Bristol, UK) were added to the Tyrode solution to block NMDA and GABA_B_ receptors, respectively. The standard intracellular solution was (in mM): 90 CsCl, 20 tetraethylammonium (TEA)-Cl, 10 EGTA, 10 glucose, 1 MgCl_2_, 4 ATP, 0.5 GTP and 15 phosphocreatine, pH 7.4. K^+^ was substituted for Cs^+^ and TEA^+^ in the pipette solution to block outward K^+^ currents. QX-314 (*N*-2,6-dimethylphenyl carbamoylmethyl triethylammonium bromide; 10 mM; Tocris) was added to block Na^+^ currents when extracellular stimulations were used. To analyze the paired-pulse ratio (PPR), two brief extracellular depolarizing pulses were applied to the presynaptic neuron at time intervals ranging between 40 ms and 10 s. For each couple of ePSCs, the PPR was calculated as I_2_/I_1_, where I_1_ and I_2_ are the amplitudes of the PSCs evoked by the conditioning and test stimuli, respectively [46]. Because of the high intrinsic variability of PPR, the mean PPR was averaged from at least five paired-pulse stimulations for each interpulse interval.

**Estimation of Pr and ensemble RRP size by cumulative amplitude analysis:** The release probability Pr was estimated applying the cumulative amplitude analysis, previously used in cultured neurons [47], [48], [49] *Calix* of Held synapses [50] and hippocampal slices [51]. High frequency stimulation (1 s @ 40 Hz) was applied to presynaptic fibers with an extracellular electrode. The analysis assumes that the depression during the steady-state induced by the train is limited by a constant recycling of SVs and that equilibrium is present between released and recycled SVs. The number of data points to include in the fit of the steady-state phase was evaluated by calculating, for each cell, the best linear fit which included the maximal number of data points starting from the last one. According to this procedure, the Y-intercept yielded an estimation of the size of the synchronous readily releasable pool (RRP) and the ratio of the first PSC evoked by the stimulation train to the RRP size yielded an estimation of Pr. When this form of quantal analysis is applied to synaptic currents evoked by the minimal stimulation method, the RRP size (i.e. the total RRP of the synapses activated by the stimulus) can be potentially affected by differences in the number of activated fibers. For this reason, this parameter was named as ensemble RRP. On the contrary, the estimation of Pr is substantially independent of changes in the number of stimulated fibers.

**MEA recordings:** Spontaneous and evoked activity expressed by random and bio-patterned cultures on MEAs were acquired by using a MCS MEA 1060 system (Multi Channel Systems, Reutlingen, Germany) with a sampling frequency of 10 kHz/channel. To evoke network activity, 50 electrical stimuli were delivered at 0.2 Hz to user-selected microelectrodes with a stimulus generator MCS STG 1008 (Multi Channel Systems, Reutlingen, Germany), which delivered biphasic rectangular voltage pulses of 1.5 V peak-to-peak in amplitude and 260 µs duration per phase. Recordings were performed at 37°C using a TC0_2_ temperature controller (Multi Channel Systems, Reutlingen, Germany). Experiments on MEAs were performed at 14 and 21 DIV on both patterned (n = 6) and random (n = 6) cultures. In order to ensure a homogeneous patterned area on the 8×8 MEAs and the correspondence between electrodes and grid's hubs, the grid was printed on a larger surface, typically in the range of 2.5×2.5 mm^2^.

**Analysis of MEA recordings:** Raw data acquired by MEAs were spike detected by using the Precise Timing Spike Detection (PTSD) algorithm [52]. The mean firing rate (MFR) and the mean bursting rate (MBR) were computed by custom scripts [19] in Matlab (The MathWorks, Natick, MA, USA). All statistical tests were conducted by using the Mann-Whitney and Kruskal-Wallis non-parametric tests [53]. Functional connectivity (FC) maps were built by cross correlating spontaneous spike events among all electrode pairs [54]. Each cross correlation was normalized by the factor (N_x_N_y_)^1/2^ where *N_x_* and *N_y_* correspond to the recorded spikes of the cross correlated x and y electrodes. The strength of each link was then defined as the normalized CC-peak value. Links were ranked based on the defined strength such that the selection of N links corresponded to the strongest N links. The statistical significance of cross correlation values was verified by using surrogate methods for correlation analysis [55]. In particular, we used the spike time dithering technique, with dither window size equal to mean interspike intervals, and defined the noise threshold as the mean plus three times the standard deviations of the dithered distributions. The time lag of a CC-peak, representative of time delays involved in synaptic and cellular integration [54], was used to discard false positive links that were not plausible based on physiological constraints (i.e., propagation velocity ≤400 mm/s [56]). By selecting the strongest N links, FC was investigated by computing: (i) the amount of horizontal and vertical links (N_HV_) lying along the imposed grid; (ii) the percentage of horizontal/vertical links by normalizing N_HV_ to the total number of links; (iii) the Euclidian lengths and the CC-delays for the selected K links and (iv) the path length computed as the sum of the Euclidian link lengths of the closed path. Post Stimulus Time Histograms (PSTHs) were built by temporally aligning the evoked spike responses to the same stimulus repeated at low frequency (0.2 Hz). Evoked responses were quantified by averaging the PSTHs of the electrodes falling in a specified square area surrounding the stimulation electrode. The core of the data analysis was accomplished by custom scripts coded in Python [57] and MATLAB (The MathWorks Inc., Natick, MA, USA).

## Results

### Development of primary neurons confined to a printed grid pattern

**Figure 2:**
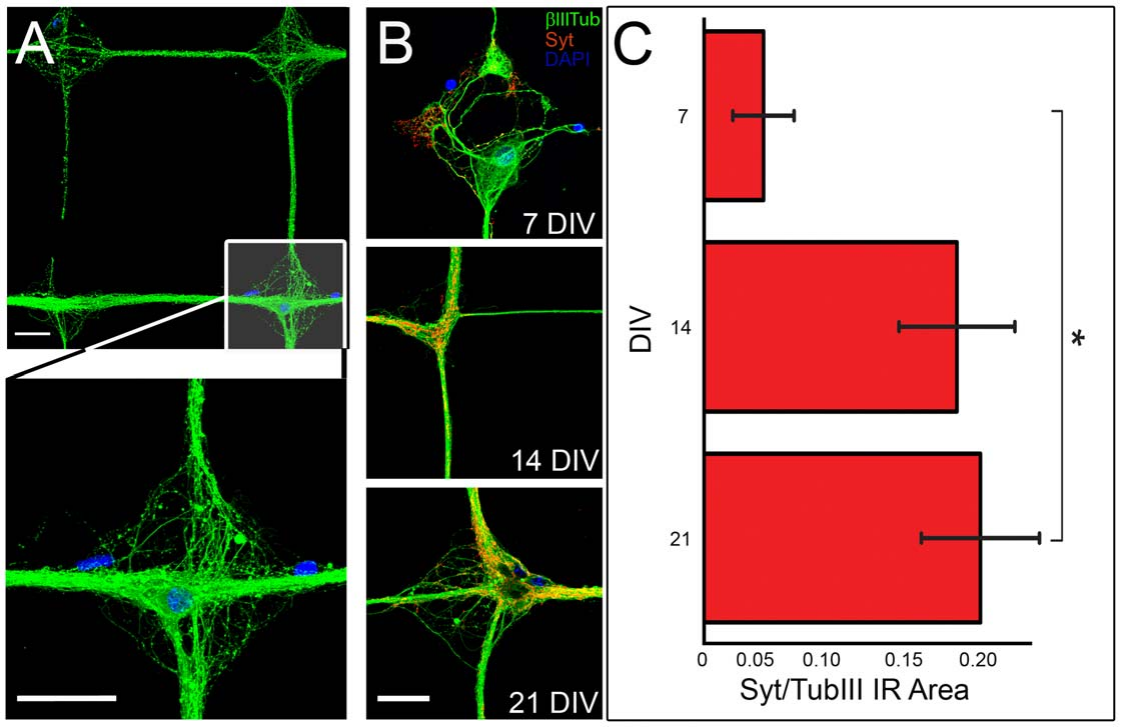
Close-ups of patterned neuronal networks and assessment of synaptic development. *(A)* Close-up of a 21 DIV culture on a grid mesh (*top*) and on a node (*bottom*). A well established and organized wiring is visible with neuronal cell bodies predominantly localized at the nodes. *(B)* Synaptotagmin (*Syt*) staining performed at 7, 14 and 21 DIV reveals the formation of synaptic contacts concentrated on the neuronal cell bodies and proximal dendrites at the nodes. *(C)* Immunofluorescence images for βTubIII or Syt were converted into a binary format and the respective immunoreactive areas corresponding to the total neuronal and synaptic areas, respectively, were quantified. The formation of synaptic connections over time was assessed by calculating the ratio between the Syt and the βTubIII immunoreactive areas. The progressive increase of the *Syt/*β*TubIII* ratio at 7 DIV (n = 3, 4 fields/sample), 14 DIV (n = 2, 4 fields/sample) and 21 DIV (n = 3, 5 fields/sample) indicates the progressive formation of synaptic connections and maturation of the neural network. Scalebar: *A* and *B*, 25 µm.

We achieved preservation of the bio-printed network topology for more than 20 DIV, as required for functional studies on mature neuronal networks. An overview of the micro-contact tools for bio-printing is illustrated in Fig. 1. As shown in the optical and Environmental Scanning Electron Microscope (ESEM) pictures (Fig. 1*A,B*), the topographical features of the grid pattern were well replicated from the original Si-etched master into the PDMS stamps. Printing of FITC-labelled PLL on a glass coverslip confirmed the efficient and homogeneous transfer of the grid pattern to the culture substrates (Fig. 1*C*). Identical printing results were obtained on agarose coated substrates (not shown). When neurons were grown on uncoated patterned coverslips, the adhesive constraint was very effective at early stages of development (up to 7–14 DIV), but this was not sufficient to confine neurites on tracks for longer times (Fig. 1*D*, *right panel*). On the contrary, the topological confinement was effectively preserved for up to 3 weeks on agarose-coated substrates (Fig. 1*D*, *left panel*). The proper development of neurites in neurons from patterned cultures was assessed by cell transfection and electrophysiology experiments (Supporting Information S1). Over *in vitro* development, synaptic connections mainly formed at the nodes of the grid pattern (Fig. 2, *A* and *B*). The formation of synaptic connections was quantitatively assessed by calculating the ratio between the immunoreactivity for the synaptic vesicle marker Syt and the total neuronal area immunoreactive for βTubIII. In the bio-printed networks, synaptic development dramatically increased from 7 to 14 DIV and subsequently stabilized (Fig. 2*C*). This developmental pattern was closely similar to what observed in random cultures using either presynaptic or postsynaptic markers [58], [59].

### Synaptic transmission and short-term plasticity in patterned networks of primary neurons

**Figure 3:**
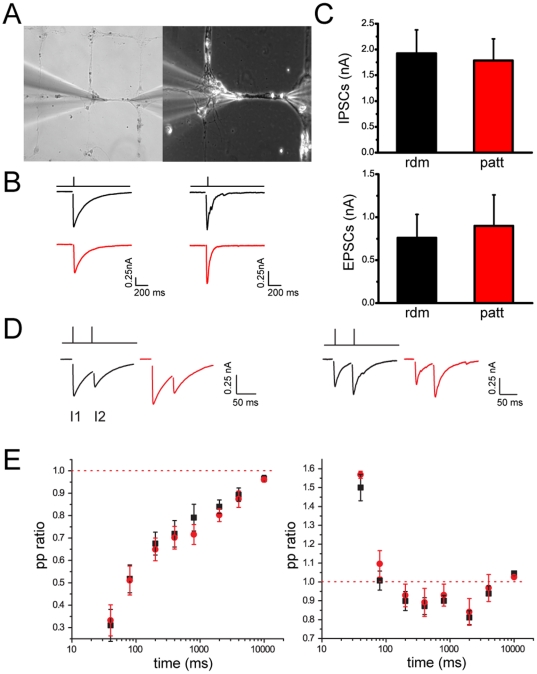
PSCs evoked in random (*rdm*; black) and patterned (*patt*; red) neuronal cultures show the same amplitude, kinetics and paired-pulse responses. *(A)* Bright field (*left*) and phase contrast (*right*) image of hippocampal neurons plated on grid topologies. *(B)* IPSCs (*left*) and EPSCs (*right*) evoked in neurons plated under either random or patterned conditions. The typical kinetics of eEPSCs and eIPSCs were not affected by the patterning procedure. *(C)* eIPSC (GABA; *top panel*) and eEPSC (GLU; *bottom panel*) amplitude (means ± SE) recorded in neurons plated under the two conditions (IPSCs: *rdm n* = 8, *patt n* = 6; EPSCs: *rdm n* = 7, *patt n* = 5). *(D)* Paired-pulse stimulation induced synaptic depression (PPR<1) in inhibitory synapses (*left traces*) and synaptic facilitation (PPR>1) in excitatory synapses (*right traces*). *(E)* Paired-pulse ratios measured at increasing interpulse intervals for GABAergic (*left panel*) and GLUergic (*right panel*) PSCs recorded from neurons plated under random (n = 5) and patterned (n = 5) conditions. Data are shown as means ± SE. All recordings were performed at a holding potential of −70 mV.

Patch-clamp recordings were performed to assess functional differences in excitatory and inhibitory synapses between mature patterned and random networks (14–21 DIV). On bio-printed networks, neuronal structures were easily identifiable and accessible to the recording pipette (Fig. 3*A*). EPSCs and IPSCs evoked by extracellular stimulation in random and bio-printed networks were perfectly comparable in terms of both kinetics and mean amplitudes (Fig. 3, *B* and *C*). In both random and patterned cultures treated with CNQX, monosynaptic ISPCs could be easily evoked and showed similar decays (τ_decay_ = 30.9±2.2 msec, n = 7 and τ_decay_ = 28.4±2.4 msec, n = 8 for random and patterned neurons, respectively). On the contrary, random cultures treated with bicuculline, typically showed EPSCs characterized by multiple EPSCs peaks, attributable to the reverberant stimulation of polysynaptic excitatory connections, even if minimal extracellular stimulation was used (see Fig. 3B). Interestingly, when extracellular stimulation was performed on patterned samples, the decaying time constant was significantly faster (τ_decay_ = 11.6±1.6 msec, n = 6 and τ_decay_ = 6.7±1.3 msec, n = 8 for random and patterned neurons, respectively; p<0.05 Mann-Whitney U test). This result suggests that the reverberation of excitatory inputs was reduced in patterned cultures, allowing to sample monosynaptic ESPCs more easily.

**Figure 4:**
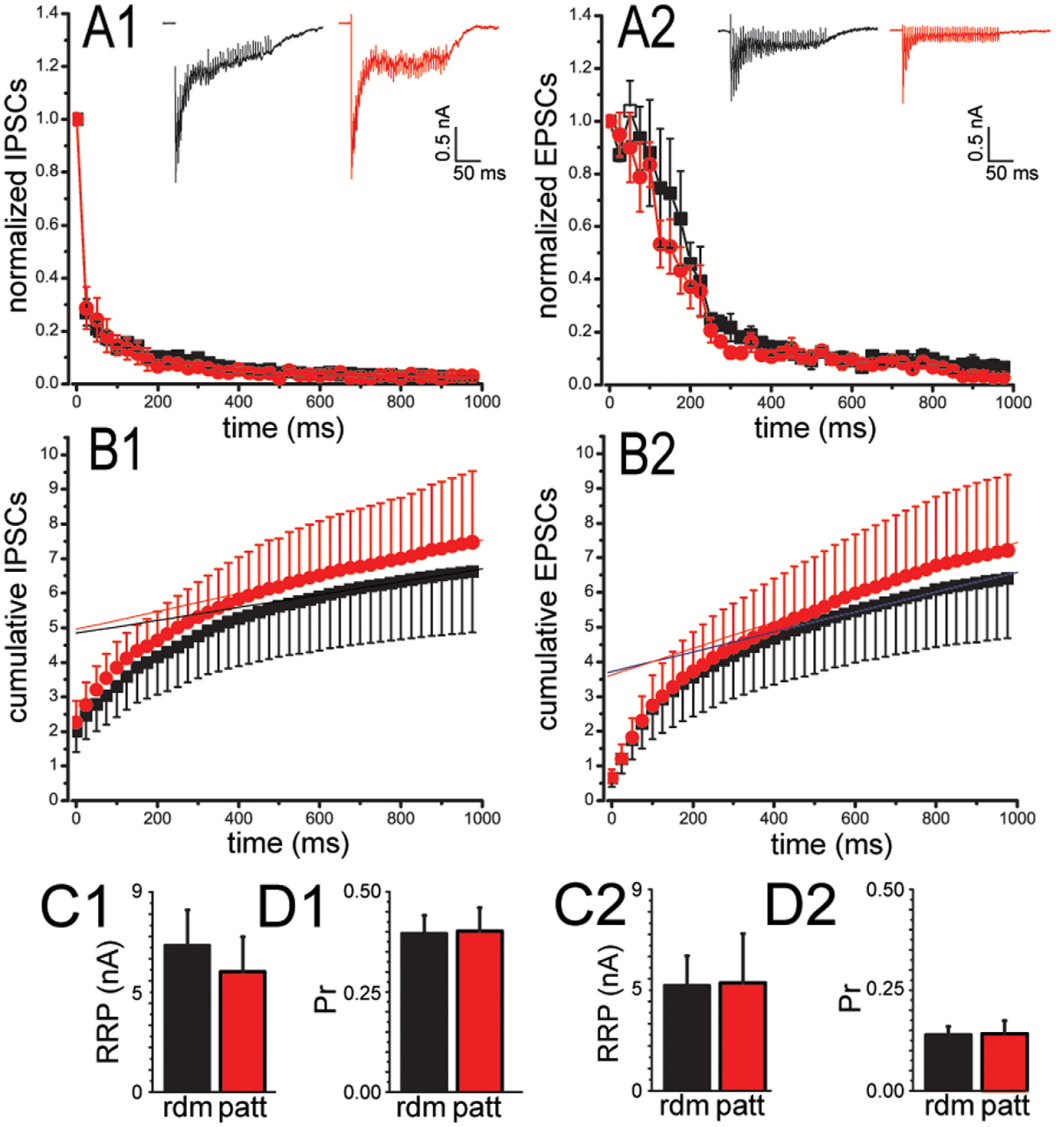
No changes in the size of the RRP and Pr of excitatory and inhibitory synapses were observed between random (*rdm*; black) and patterned (*patt*; red) cultures. *(A1, A2)* Plots of the PSC amplitude (means ± SE) vs time during train stimulation of GABAergic (*A1*; *rdm n* = 6, *patt n* = 9) and glutamatergic neurons (*A2*; *rdm n* = 4, *patt n* = 7) plated under random or patterned conditions. Representative currents evoked by high frequency stimulation (1 s @ 40 Hz) applied to GABAergic and glutamatergic neurons plated at low density under random or patterned conditions are shown in the respective insets. *(B1, B2)* Cumulative IPSC (*B1*; *rdm n* = 4, *patt n* = 4) and EPSC (*B2*; *rdm n* = 4, *patt n* = 4) profiles for random and patterned neurons. Cumulative analysis evaluation (means ± SE) of the ensemble RRP size *(C1,C2)* and of the Pr *(D1,D2)* for GABAergic (*C1,D1*) and glutamatergic (*C2,D2*) synapses from random and patterned neurons. Statistical analysis was carried out by using the Mann-Whitney U test. *, *p*<0.05 vs *rdm* group (GLUergic neurons, *n* = 6 and *n* = 8 for random and patterned neurons, respectively; GABAergic neurons, *n* = 5 and *n* = 8 for random and patterned neurons, respectively). All recordings were performed at a holding potential of −70 mV.

We next investigated short-term plasticity properties evoked by specific patterns of presynaptic stimulation, such as paired stimuli or high frequency trains. Paired stimuli were applied to random or patterned hippocampal neurons at increasing inter-pulse intervals ranging from 25 ms to 10 s (Fig. 3*D*). Control inhibitory synapses responded to the second stimulus with a depression in the PSC, which was very intense at short interpulse intervals and progressively attenuated at longer times. On the contrary, control excitatory synapses displayed a marked increase of the PSC at short interpulse intervals, defined as facilitation, which vanished at longer times (Fig. 3*E*). These opposite responses, attributable to different levels of release probability, were fully preserved when neurons were grown under patterned conditions, indicating that the basic mechanisms of evoked neurotransmitter release were not affected by the topological confinement. To further investigate the effects of patterning on the quantal parameters of release, namely the release probability (Pr) and the size of the ensemble ready releasable pool (RRP), we analyzed the cumulative amplitude profile during high frequency trains of stimuli applied (1 s at 40 Hz) applied to either GABAergic or glutamatergic neurons (Fig. 4). A significant depression of PSCs became apparent during the trains in both inhibitory and excitatory synapses. Such depression was faster in GABAergic (Fig. 4*A* *1*) than in glutamatergic (Fig. 4*A* *2*) synapses, but the temporal profiles of depression were closely superimposable under either random or patterned culturing conditions. In both random and patterned groups, the cumulative profile of repeated eIPSCs or eEPSCs showed a rapid rise followed by a slower linear increase of different steepness at later pulses (Fig. 4*B*). Both the ensemble RRP (Fig. 4*C*) and the Pr (Fig. 4*D*) were not affected by the culturing conditions, with inhibitory synapses showing typically higher Pr values than excitatory synapses consistent with the results of the paired-pulse experiments.

### Activity and functional connectivity in patterned networks of primary neurons grown on MEAs

**Figure 5:**
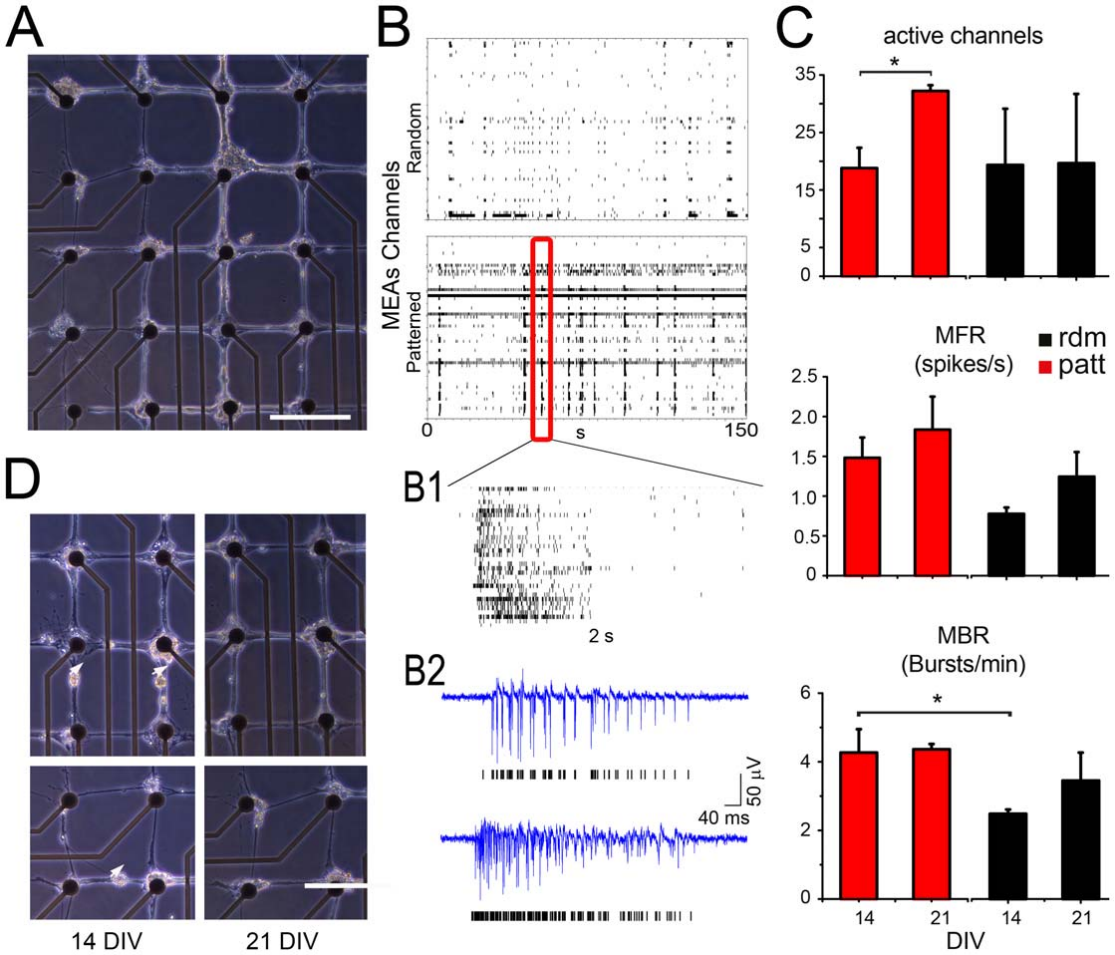
Analysis of the network activity emerging from grid-patterned neuronal populations coupled to MEAs. *(A)* Microphotograph of a typical bio-patterned network aligned with the electrode array. *(B)* Raster plot of basal extracellular activity (time window, 150 s) on random (*upper panel*) and printed (*bottom panel*) cultures. On the right *(B1)*, a close-up (time window, 2 s) of the firing electrodes. *(B2)* representative traces of two synchronously firing channels. *(C)* Comparison of electrophysiological features of two sample groups (*Red*, *Patterned n* = 5; *Black Random* n = 5) at 14 and 21 DIV. While the MFR did not significantly change, the number of active channels (threshold = 0.1 spike/s) was significantly increased and the MBR was significantly higher at 14 DIV in patterned samples. *(D)* Example of the migration of neuronal somas to the grid's nodes, favouring cell localization in close proximity to the electrodes in mature networks. Scale bars: 200 µm.

We next performed experiments on MEAs to investigate whether the imposed grid topology was correlated with the emerging activity expressed at the network scale. An example of a bio-printed culture with a grid pattern aligned to the MEA array is shown in Fig. 5*A*. The overall network activity at 21 DIV (Fig. 5*B*) revealed the expression of a typical bursting behaviour similar to that of random high density cultures [60]. At early stages of maturation (14 DIV), the extent of synchronisation was precociously high in bio-patterned networks, while only rare and occasional bursts could be observed in random low-density cultures. However, once full network maturation was reached (21 DIV), both types of cultures showed similar firing and bursting rates (Fig. 5*C*), suggesting that the overall network activity was not significantly modified by the imposed grid topology. Interestingly, the number of active electrode channels (threshold = 0.1 spike/s) was stable (*p* = 0.67, Mann-Whitney U test) over development in random cultures, whereas it greatly increased over time in patterned cultures (*p*<0.01, Mann-Whitney U test). Such an increase denotes an improvement of the neuron-electrode coupling during maturation of the bio-printed network and could be contributed by the observed migration of neuronal cell bodies to the nodes of the grid corresponding to the microelectrodes, where larger adhesion sites are available (Fig. 5*D*).

**Figure 6:**
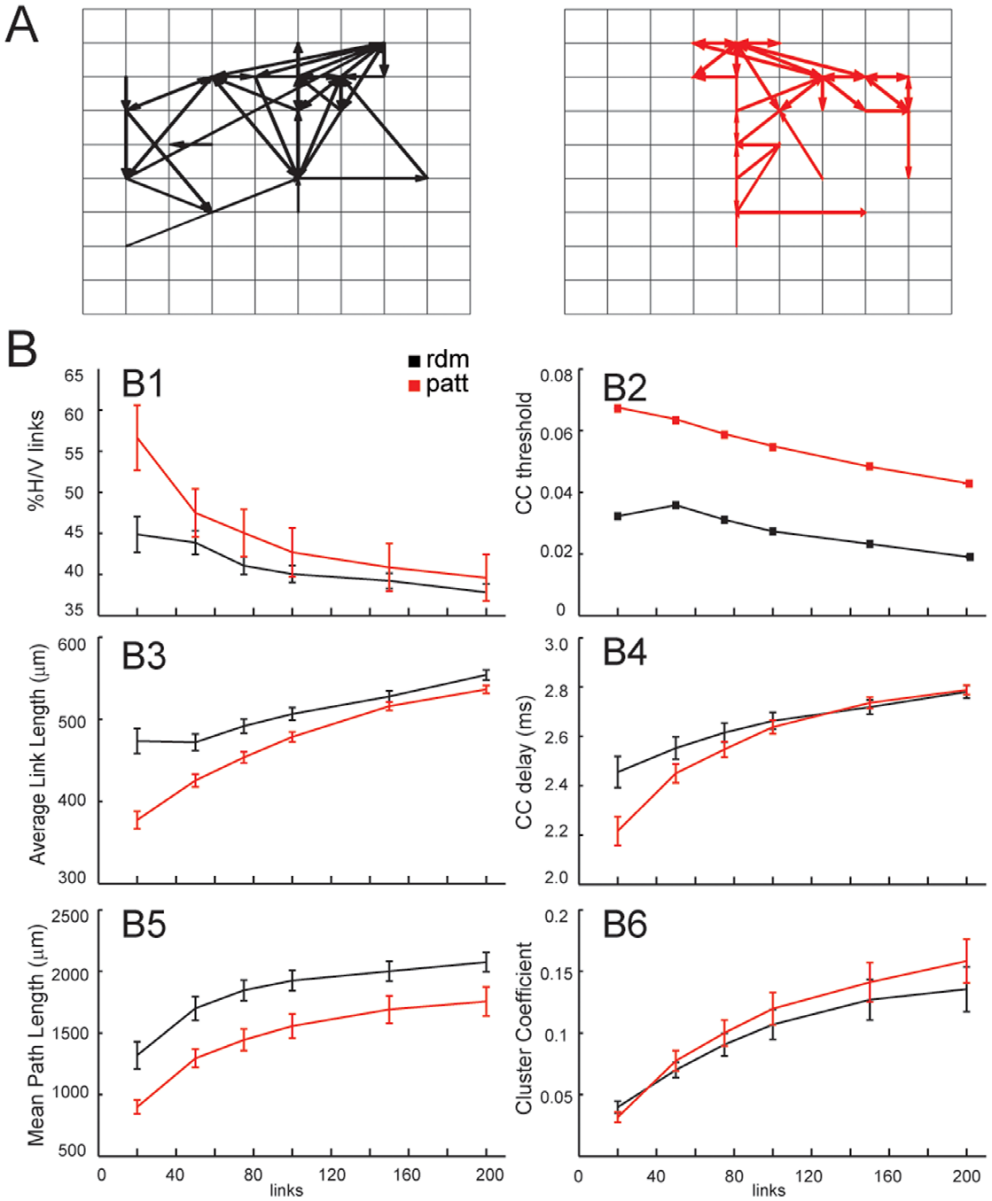
Functional connectivity (FC) analysis on 21 DIV grid (red, n = 26) and random (black, n = 19) cultures grown on MEAs. *(A)* FC maps of patterned (*right panel*) and random (*left panel*) cultures obtained by selecting the strongest 50 cross-correlation links. *(B)* By varying the number of strongest links, the following FC parameters were estimated: percentage of horizontal/vertical links *(B1)*; cross-correlation value of the weakest link selected *(B2)*; average link length *(B3)*; cross-correlation delays *(B4)*; mean pathlength *(B5)*; clustering coefficient *(B6)*. The strongest links (up to ∼50 links) in patterned MEAs displayed significantly shorter lengths and smaller CC-delays than random cultures, compatible with the imposed geometry.

To ascertain whether the functional connectivity was affected by the imposed topology, we analyzed connectivity maps in patterned and random MEA cultures (Fig. 6). Functional connectivity maps of the 50 strongest links, i.e. those with the highest cross-correlation peaks, on the patterned samples revealed spatially-ordered connections reflecting the imposed topology (Fig. 6*A*). To quantify these differences, we computed the percentage of vertical and horizontal links (v/h), cross-correlation time delays (CC delay), average link lengths and average pathlengths (Fig. 6*B*) at 21 DIV (n = 26 random, n = 19 patterned). A significant difference in the percentage of v/h versus diagonal links was found (Fig. 6*B* *1*) up to 50 links, indicating an emerging directional preference of the links corresponding to the spatially-imposed connectivity. The analysis was limited to the strongest 200 links (i.e., 60% of the total links) in order to consider only significant links overcoming the noise threshold for the normalized cross-correlation of random cultures (Fig. 6*B* *2*). The average link length and CC-delays (Fig. 6,*B3* and *B4*) showed lower values for the grid-patterned networks with respect to the random culture condition due to a functional reinforcement of the network connectivity along the imposed horizontal and vertical directions. Paths across the network formed by consecutive links were quantified by the mean pathlength (Fig. 6*B* *5*). In line with the observed lower average link length, the mean pathlength was significantly lower in grid networks as compared to the random ones (Mann-Whitney test, p<0.05). Interestingly, the clustering coefficient was low and showed a comparable trend on both topologies (0.2 up to 200 links; Fig. 6*B* *6*), indicating that both topologies display similar network connectivity properties, in spite of the different link organization.

**Figure 7:**
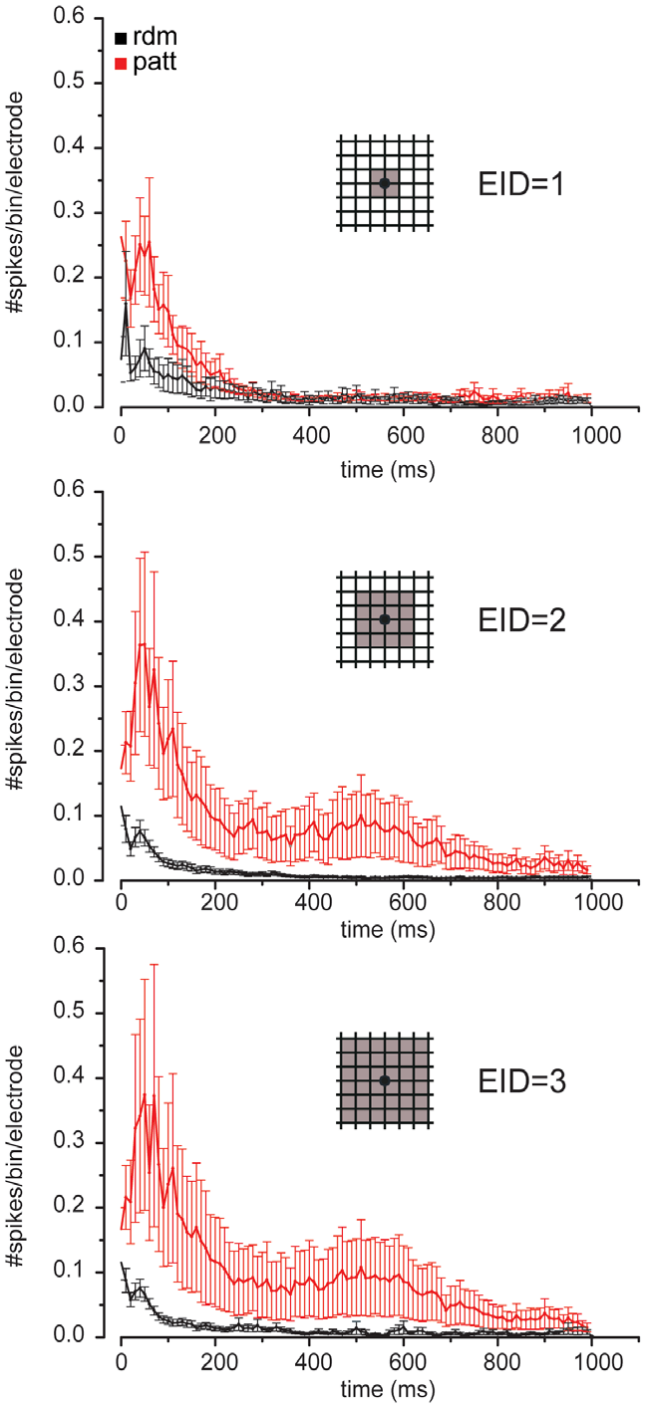
Post-Stimulus Time Histograms (PSTH) computed on random (*black*, n = 9) and patterned (*gray*, n = 6) cultures. Electrical stimulation of patterned MEAs elicited a higher number of spikes at various distances from the stimulation electrode. Increasing square areas around the stimulation electrode were analyzed. At electrode inter distance (*EID*) = 1, the differences were evident up to 200 ms. When patterned versus random cultures were compared at EID = 2 and EID = 3, the difference was systematic on the entire time interval, while PSTH shapes were quite similar. At different EIDs, the average number of spikes increased in patterned cultures, while it remained rather constant in random cultures.

Finally, the effect of topology on the propagating responses evoked by electrical stimulation was assessed by computing the PSTHs and by varying the size of the recording areas surrounding the stimulation electrode (Fig. 7). In patterned networks, the probability of detecting evoked spikes increased with the recording area. The shape of the PSTH curves was quite similar for the various areas and higher PSTH areas were found on grid topologies, likely due to shorter links and pathlengths.

Interestingly at the smallest inter-electrode distance (EID = 1, i.e., where the recording electrodes fall close to the stimulation one; Fig. 7), the PSTH curve differed and lasted only up to ∼300 ms. Thus, the PSTH analysis denotes that a higher probability of detecting short range connections was found in patterned networks, in which short range connections are forced and aligned with the electrode array.

## Discussion

Topologically-defined neuronal networks grown on MEAs offer valuable experimental opportunities for investigating the interplay between anatomical and functional connectivity and facilitate morphological, electrophysiological and computational investigations on large neuronal assemblies in vitro. Here, the development of an effective bio-printing methodology was applied for the first time to maintain a defined topology in neuronal networks over time in culture and combined with patch-clamp and MEA recordings to investigate the resulting functional properties of the patterned network. We demonstrated that the use of micro-contact printed adhesive surfaces ECM-PDL onto a repulsive agarose layer sustains a reliable topological confinement of neurons by selectively enhancing cell adhesion within the printed pattern and inhibiting cell growth outside it. Under these conditions, low-density preparations of primary hippocampal neurons were successfully grown for more than 21 DIV, reaching the stage of mature synaptically-connected networks expressing both spontaneous and evoked activities.

Random and grid topologies displayed rather similar properties in terms of synapses formation and network maturation. Patch-clamp analysis showed that both excitatory and inhibitory synapses in patterned and random networks displayed comparable kinetics and amplitudes, as well as closely similar quantal parameters of release and short-term plasticity responses to paired stimuli and high frequency trains. Interestingly, we observed that after extracellular stimulation of patterned cultures, the probability to recruit monosynaptic EPSCs without multiple EPSC peaks in the decay phase was greatly increased. This represents a clear advantage offered by the patterning technique with respect to random cultures. These results demonstrate the neurophysiological reliability of our bio-printed grid networks and support further investigations on MEAs at the population scale.

An interesting question that we investigated is whether the specific grid topology triggers any specific functional property at the network scale. Interestingly, simple statistics of network activity in patterned and random cultures showed an overall comparable development of synaptic connections and a similar inhibitory/excitatory ratio. In fact, both types of networks reached similar rates of spontaneous firing and bursting upon network maturation, although synchronized bursting activity appeared more precociously in patterned networks. The significant increase in the number of active electrodes observed in mature printed networks resulted from the preferential location of neuronal cell bodies at the electrode and from the improvement of the neuron-electrode coupling.

While this analysis did not show significant differences between mature networks of the two topologies, functional connectivity analysis showed that stronger links with shorted delays and lengths emerged along vertical and horizontal directions in grid networks. This observation is in line with what can be expected by assuming an ideal grid topology, characterized by a simplistic connection rule, where each electrode/neuron is connected to its nearest neighbours. Indeed, in an ideal grid, connections are ordered in vertical/horizontal links and neighbours of a given electrode are not directly connected among themselves. Thus, under these conditions, an ideal grid topology would share some properties with random and regular networks [61], respectively characterized by a null clustering coefficient and a high mean path length (much higher than in random networks).

The functional connectivity analysis performed on random and patterned networks proved that the clustering coefficient and the mean path length increased with the analyzed links up to 200 links. While the clustering coefficient was comparably low (<0.2) in both grid and random preparations the mean path length was always lower in the grid topology. This represents a peculiar property of the grid networks: being the mean path length the lowest among random, ideal grids and regular networks, electrical activities would be expected to spread more efficiently from one point to another one in the grid network. Being the mean path length markedly lower in grid networks, the distribution of the functional links does not follow the simplistic connection rule of the ideal grid topology.

On the contrary, the clustering coefficient for grid and random networks was quite comparable and the former showed only a slight tendency to increase respect to random networks. It is intriguing to compare the properties of the grid networks respect to the so called Small World ones (*SW*, [61]) characterized by low mean path length values but also by high clustering coefficient values. Because here we had to limit the analysis to a reduced set of links (200 links), we cannot yet reliably estimate the clustering coefficient of the whole network. As such we can only assess that grid networks display a tendency toward small-worldness.

Given the low spatial resolution provided by MEAs used in this work (i.e., 200 µm in electrode pitch), a significant spatial undersampling of the recorded network activity in random cultures has to be taken into account, resulting in unmeasured neurons among the electrodes. Thus, for random networks, the computed mean path length has to be considered as a lower bound evaluation. Recent advances in high resolution MEA technologies such as APS-MEA devices [62] could contribute in refining these results by increasing the number of detectable links and by effectively performing these studies at the cellular level. Moreover, the cross-correlation based methodology applied here to estimate the functional connectivity might also take advantage from different methodologies [63].

The analysis of the evoked network responses indicates a similar long-range propagation behaviour of the two topologies, while short-range connections are detected with a higher probability on grid networks than on random networks. Thus, by imposing and aligning the network topology on MEAs, it was possible to achieve a higher spiking probability in response to the stimulation and an improved propagation of evoked signals at both short- and long-range scales.

Overall, the core study of grid networks shows that they retain key electrophysiological features with a rather similar behaviour to random preparations, despite the distinct functional and overall anatomical connectivity. Interestingly, our grid topology is not impeding long-range connections among multiple nodes, but it is effectively confining the network within the imposed topology. Interestingly, the extensions of single-neuron neurites in patterned networks reached an average length of 625±81 µm that was confirmed by parallel electrophysiological estimations based on cable theory (see Supporting Information S1). These results confirm theoretical considerations on connectivity parameters for both topologies. This case study on grid topology represents also an important methodological step for understanding how an altered anatomical connectivity influences the emergent network activity, as well as for implementing biologically-inspired topologies which encode predesigned computational properties.

## Supporting Information

Supporting Information S1 **Analysis of the average neurite extension of mCherry-transfected neurons grown in grid patterns.** The average neurite length was evaluated by transfecting a limited number of neurons in the network with mCherry. The transfection protocol demonstrates that the imposition of the pattern did not prevent neurites to develop normally as in standard random cultures. Fig. S1 (left panel) shows that the transfection protocol allowed to effectively track neurites of a single neuron up to 800 µm from the soma. The quantification yielded to an average neurite length of 625±81 µm (n = 4).
(DOCX)
