# Supplementary material for: Emergent Functional Properties of Neuronal Networks with Controlled Topology
Source: PLoS One. 2012 Apr 6;7(4):e34648. doi: 10.1371/journal.pone.0034648 (PMC3321036; doi:10.1371/journal.pone.0034648)
Supplement: Supporting Information S1 — Analysis of the average neurite extension of mCherry-transfected neurons grown in grid patterns. The average neurite length was evaluated by transfecting a limited number of neurons in the network with mCherry. The transfection protocol demonstrates that the imposition of the pattern did not prevent neurites to develop normally as in standard random cultures. Fig. S1 (left panel) shows that the transfection protocol allowed to effectively track neurites of a single neuron up to 800 µm from the soma. The quantification yielded to an average neurite length of 625±81 µm (n = 4). (DOCX) [file pone.0034648.s001.docx]

Supporting Information

*Analysis of the average neurite extension of mCherry-transfected neurons grown in grid patterns*

The average neurite length was evaluated by transfecting a limited number of neurons in the network with mCherry. The transfection protocol demonstrates that the imposition of the pattern did not prevent neurites to develop normally as in standard random cultures. Fig. S1 (left panel) shows that the transfection protocol allowed to effectively track neurites of a single neuron up to 800 μm from the soma. The quantification yielded to an average neurite length of 625±81 μm (n=4).

| 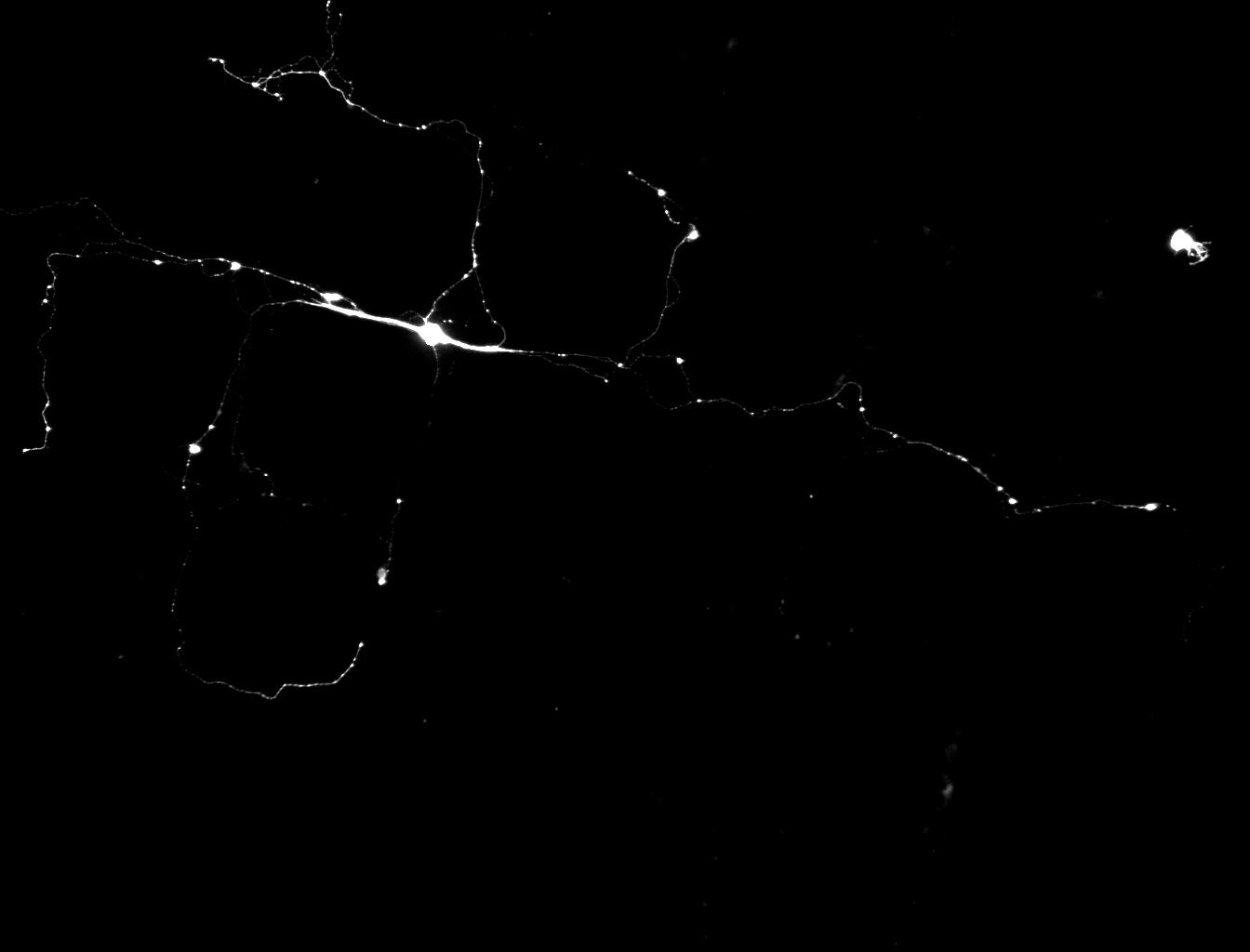 | 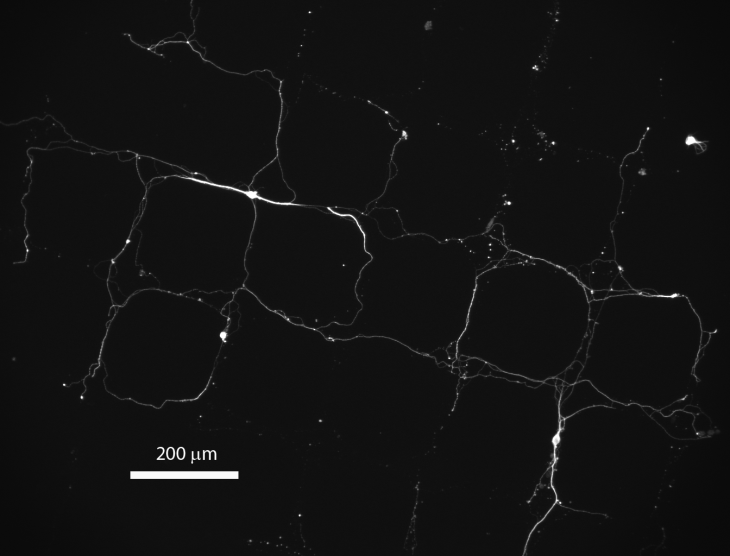 |
| --- | --- |
| **Figure S1.** AMAXA transfection of a single neuron. (Left) An isolated soma (bright circle in the upper left part of the figure) has neurites extending in all directions permitted by the imposed pattern. The grid structure of the pattern, quite noticeable in the figure, reflected that of the same geometry imposed on the MEA recording system with an inter-node spacing of 200 μm. Consequently, here we show that neurites can extend significantly from the soma over multiple nodes, up to 800 μm (4 nodes). (Right) The same culture was counterstained with an antibody against βTubIII to show the whole network development. The two images are reported on a different scale to provide a detail of the extensions of a single transfected neuron (left) and to image additional non-transfected neurons in the network (TubIII, right) . | |

*Methods.* E18 rat primary hippocampal neurons were prepared as described in the Material and Methods section. The transfection was performed using Amaxa 4D-Nucleofector X Unit (Lonza, Cologne, Germany) following the manufacturer’s protocol for hippocampal^(1)^ neurons. Cells were transfected with the program CU-133 and 1.5 µg pCAGGS vector containing mCherry red fluorescent protein [1]. In order to enable fluorescence imaging of a few neurons, the transfected cellular suspension was diluted 1:2 with non-transfected cellular suspension.

*Estimation of neurite extension of single neurons grown in grid patterns by cable theory.*

In addition to the AMAXA transfection protocol, we also performed electrophysiological experiments to assess the neurite extension. This independent electrophysiological analysis was in agreement with the morphological evaluations and confirmed the presence of functional long neurites.

We have determined the passive membrane properties of random and patterned neurons (> 14 DIV) following the protocol devised by Rall [2,3]. Briefly, short current pulses (0.5 ms) were injected into the neurons and the decay phase was fitted to the sum of a series of exponential curves. To limit the involvement of voltage-activated channels, hyperpolarizing pulses (ranging from -100 pA to -500 pA, stepped by 100 pA) were mainly used.

The fitting procedure was carried out according to the optimization procedure described in [2] that allows to determine the components of the multi-exponential decay more efficiently as compared to the classical ‘*peeling*’ technique. The fit allowed us to compute the *electrotonic length* (Eq. 3 in [2]) and the *input resistance* (Eq. 1 in [2]). For all tested currents, both parameters were quite comparable between random and patterned neurons.

As an example, the ‘*peeling’* protocol (pulses of -400 pA) yielded no significant differences either in the input resistance (R_in_=144.4±37.7 and 209.5±31.6 MΩ in neurons from patterned [n=5] and random [n=6] cultures, respectively; p=0.31, Student’s t test) or in the electrotonic length (L=1.13±0.53 and 1.44±0.38 in neurons from patterned [n=5] and random [n=6] cultures, respectively; p=0.65, Student’s t test). For comparison, in hippocampal dentate granule cells D’Aguanno et al. [1] obtained L=1.51 that was quite comparable with our results.

The cable theory allows to estimate the neurite extension (*ne*) knowing the length constant (*λ*), by equation: *ne = Lλ*. The length constant is further related to the diameter of the neurite (*d*), the membrane resistance (*R_m_*) and the axial resistance (*R_i_*) by the equation $\text{}\text{ }\text{=}\sqrt{{d \cdot R}_{m}/{(4\cdot R}_{i})}$. Then assuming R_m_=20 KΩ cm^2^ , R_i_= 100 Ω cm [3] and a dendrite of 1 μm, the length constant would be $\cong$ 700 μm and the neurite extension *ne*$\cong$ 700 · 1.44 $\cong$1 mm, that results in dendrites spanning up to 4-5 nodes. This result is in agreement with the data obtained from mCherry-transfected neurons and with similar determinations performed in hippocampal principal cells [4].

References:

1. Niwa H, Yamamura K, Miyazaki J (1991) Efficient selection for high-expression transfectants with a novel eukaryotic vector. Gene 108:193-199
2. D’Aguanno et al. IEEE Trans Biomed Eng. 33:1188-1196,1986
3. W.Rall, H.Agmon-Snir “*Cable Theory for Dendritic Neurons*” in: *Methods in Neuronal Modeling* C.Koch, I.Segev ed., The MIT Press
4. The Hippocampus Book, edited by P. Andersen, R. Morris, D. Amaral, T. Bliss and J. O'Keefe, Oxford University Press (2007)
